# Reducing prostate biopsies and magnetic resonance imaging with prostate cancer risk stratification

**DOI:** 10.1002/bco2.146

**Published:** 2022-04-22

**Authors:** Petter Davik, Sebastiaan Remmers, Mattijs Elschot, Monique J. Roobol, Tone Frost Bathen, Helena Bertilsson

**Affiliations:** ^1^ Department of Clinical and Molecular Medicine NTNU ‐ Norwegian University of Science and Technology Trondheim Norway; ^2^ Department of Urology St. Olav's Hospital Trondheim Norway; ^3^ Department of Urology, Erasmus MC Cancer Institute University Medical Center Rotterdam Rotterdam The Netherlands; ^4^ Department of Circulation and Medical Imaging NTNU ‐ Norwegian University of Science and Technology Trondheim Norway; ^5^ Department of Radiology and Nuclear Medicine St. Olav's Hospital Trondheim Norway

**Keywords:** biopsy, European Randomized Study of Screening for Prostate Cancer risk calculators (ERSPC RCs), magnetic resonance imaging, MRI, prediction model, prostate cancer, risk calculator, risk stratification

## Abstract

**Objectives:**

To recalibrate and validate the European Randomized Study of Screening for Prostate Cancer risk calculators (ERSPC RCs) 3/4 and the magnetic resonance imaging (MRI)‐ERSPC‐RCs to a contemporary Norwegian setting to reduce upfront prostate multiparametric MRI (mpMRI) and prostate biopsies.

**Patients and Methods:**

We retrospectively identified and entered all men who underwent prostate mpMRI and subsequent prostate biopsy between January 2016 and March 2017 in a Norwegian centre into a database. mpMRI was reported using PI‐RADS v2.0 and clinically significant prostate cancer (csPCa) defined as Gleason ≥ 3 + 4. Probabilities of csPCa and any prostate cancer (PCa) on biopsy were calculated by the ERSPC RCs 3/4 and the MRI‐ERSPC‐RC and compared with biopsy results. RCs were then recalibrated to account for differences in prevalence between the development and current cohorts (if indicated), and calibration, discrimination and clinical usefulness assessed.

**Results:**

Three hundred and three patients were included. The MRI‐ERSPC‐RCs were perfectly calibrated to our cohort, although the ERSPC RCs 3/4 needed recalibration. Area under the receiver operating curve (AUC) for the ERSPC RCs 3/4 was 0.82 for the discrimination of csPCa and 0.77 for any PCa. The AUC for the MRI‐ERSPC‐RCs was 0.89 for csPCa and 0.85 for any PCa. Decision curve analysis showed clear net benefit for both the ERSPC RCs 3/4 (>2% risk of csPCa threshold to biopsy) and for the MRI‐ERSPC‐RCs (>1% risk of csPCa threshold), with a greater net benefit for the MRI‐RCs. Using a >10% risk of csPCa or 20% risk of any PCa threshold for the ERSPC RCs 3/4, 15.5% of mpMRIs could be omitted, missing 0.8% of csPCa. Using the MRI‐ERSPC‐RCs, 23.4% of biopsies could be omitted with the same threshold, missing 0.8% of csPCa.

**Conclusion:**

The ERSPC RCs 3/4 and MRI‐ERSPC‐RCs can considerably reduce both upfront mpMRI and prostate biopsies with little risk of missing csPCa.

## 1 INTRODUCTION

The diagnostic work‐up of men suspected of having prostate cancer (PCa) includes biopsy of the prostate, which is associated with a risk of infection (1%–9.6%) and an increasing risk of life—threatening sepsis (1%–4%) due to increasing fluoroquinolone resistance.^1^, ^2^, ^3^, ^4^ Multivariable risk calculators (RCs) that use clinical parameters to predict the likelihood of finding PCa on biopsy have been developed and can be used to omit biopsy when the predicted likelihood of cancer is low. The RCs stemming from the Dutch arm of the European Randomized Study of Screening for Prostate Cancer (ERSPC) are some of the best known and validated multivariable RCs. The ERSPC RC3, for men without prior biopsy, and ERSPC RC4, for men with prior negative biopsy, can contribute to avoiding 20%–33% of unnecessary systematic transrectal ultrasound (TRUS)‐guided biopsies.^5^, ^6^, ^7^, ^8^ The ERSPC RCs 3/4 use prostate‐specific antigen (PSA), prostate volume, digital rectal examination (DRE) findings and previous biopsy history as predictors in logistic regression models that predict biopsy results. The North American Prostate Cancer Prevention Trial (PCPT)RC 2.0 is another well‐known RC that stemmed from the placebo arm of the PCPT.^9^ In a head‐to‐head comparison of the ERSPC RCs and the PCPTRC 2.0, as well as other current RCs, the ERSPC‐RCs showed superior discrimination for detecting clinically significant PCa (csPCa) as well as the greatest net benefit, followed by the PCPTRC 2.0.^7^, ^8^, ^10^ Multiparametric magnetic resonance imaging (mpMRI) of the prostate has emerged as an important investigation in PCa diagnostics due to its high negative predictive value and sensitivity in detecting csPCa^11^, ^12^ and is recommended before performing biopsy in the European Association of Urology (EAU) 2021 Prostate Cancer Guidelines.^13^ RCs that incorporate information from mpMRI have been developed, including the MRI‐ERSPC‐RCs.^14^, ^15^, ^16^ These outperform non‐mpMRI RCs,^17^, ^18^ but at the cost of performing an mpMRI examination. Prediction models need to be calibrated and validated to the population on which they will be used before implementation. No RCs have been developed or validated for Scandinavian countries. The aim of this study was to facilitate a reduction of unnecessary mpMRI examinations and prostate biopsy procedures by calibrating and validating the ERSPC RCs 3/4 and MRI‐ERSPC‐RCs in a Norwegian university hospital cohort.

## 2 PATIENTS AND METHODS

Consecutive consenting patients referred to the St. Olav's Hospital Department of Urology, Trondheim, Norway, from January 2016 to March 2017 with a suspicion of PCa were retrospectively entered into a database. Inclusion criteria were that patients had received a prostate mpMRI reported with the PI‐RADS v2.0 scoring system^19^ with subsequent prostate biopsy at our institution. Patients with a previous diagnosis of PCa and patients who did not undergo biopsy after mpMRI were excluded. The decision to biopsy a patient or not was made by the treating urologist after mpMRI. Biopsied patients generally had a PSA level > 3 ng/ml, and/or abnormal DRE, and/or PI‐RADS ≥ 3 on mpMRI.

### 2.1 Ethics statement

All patients gave informed consent; the study was approved by the institutional review board and The Regional Committee for Medical and Health Research Ethics (REC Central Norway, identifier 2017/576).

### 2.2 Imaging

All mpMRI were obtained using a 3 T MRI system (Skyra; Siemens, Erlangen, Germany) without an endorectal coil. The imaging protocol included T2‐weighted imaging in three planes, axial diffusion‐weighted imaging and dynamic contrast‐enhanced imaging, in accordance with PI‐RADS v2.0^19^ recommendations. All mpMRI were interpreted prospectively by or under the supervision of expert uroradiologists (with 2–7 years of experience in prostate mpMRI interpretation). PI‐RADS v2.0 and a standardized reporting template with a maximum of three lesions were used for all reports. All identified lesions were assigned a PI‐RADS v2.0 score for each imaging sequence and an overall PI‐RADS v2.0 score.

### 2.3 Biopsy

After mpMRI, all men underwent either systematic TRUS‐guided biopsy (*n* = 264), transrectal magnetic resonance‐guided in‐bore biopsies (MRGBs) (*n* = 35) or transperineal ultrasound‐guided cognitively targeted biopsies (*n* = 4). Nine patients underwent both systematic TRUS biopsy and MRGB as part of another study (REC Central Norway Study identifier REK2013/1869). All patients received prophylactic antibiotics in accordance with national guidelines at the time. Systematic TRUS‐guided biopsies were obtained under local anaesthesia and ultrasound guidance by a BK medical ultrasound system (Specto, BK medical, Herlev, Denmark) with a triplane transrectal probe. Biopsies were obtained in an extended core pattern consisting of two biopsies from the basis, mid‐gland and apex of each side of the prostate and one core from the central zone of each side. The treating urologists were not blinded to the findings of the preceding mpMRI. Transrectal MRGBs were performed under lidocaine 2% gel (Xylocain, Aspen Nordic, Ballerup, Denmark) anaesthesia by one of two experienced radiologists. Transperineal biopsies were obtained under general anaesthesia by the same radiologists, with cognitive ultrasound‐guided targeting in the case of PI‐RADS ≥ 3 on mpMRI. All histopathological specimens were analysed by dedicated uropathologists and graded according to International Society of Urological Pathology (ISUP) standards.^20^ A biopsy was classified as positive for any PCa with any finding of cancer and csPCa defined as Gleason score ≥ 3 + 4 (ISUP grade group ≥ 2) in any number or length of biopsies. Indolent cancers were defined as Gleason 3 + 3 (ISUP grade group 1), independent of cancer core length.

### 2.4 Statistical analyses

Missing DRE findings were imputed (MICE package for R, version 3.13.00).^21^ Prostate volumes were calculated from T2 mpMRI by the ellipsoid formula (height × length × width × π/6). The probabilities of detecting any PCa and csPCa on biopsy were calculated, using the ERSPC RC3 for biopsy naïve, the ERSPC RC4 for previously biopsied patients and the MRI‐ERSPC‐RCs, blinded to biopsy outcomes. Biopsy results were subsequently disclosed, and RCs re‐calibrated based on the intercept in the large to account for differences in prevalence between the development and validation populations. Calibration slopes were calculated and visualized in calibration plots, both from the uncalibrated and re‐calibrated RCs.^22^ Discriminative ability of the recalibrated ERSPC RC 3/4 and MRI‐ERSPC‐RCs was assessed by calculating the area under the receiver operating curves (AUC) with 95% confidence intervals (CIs) computed from 2000 bootstrap samples. Clinical utility was quantified by performing decision curve analyses (DCAs) on the recalibrated calculated probabilities for a range of threshold probabilities. DCAs give the net benefit, which is a combined measure of the benefits of identifying a true positive against the harms of undergoing an unnecessary biopsy procedure, using a given model.^23^ The number of biopsies potentially saved with the corresponding numbers of correctly identified and missed PCas and csPCas was calculated for a range (4%–20%) of threshold probabilities to biopsy. Values were standardized to 1000 patients. Subgroup analyses of biopsy‐naïve patients and of men with prior negative biopsies were performed. Sensitivity analyses excluding MRGB and cognitive targeted transperineal biopsies were performed to assess how these biopsy methods affected predictive accuracy of the RCs. Statistical analyses were performed using R version 3.5.1 (R Foundation for Statistical Computing, Vienna, Austria).

#### 2.4.1 Power calculations

On the basis of the framework of Snell et al.,^24^ we simulated the expected width of the 95% CIs of the calibration slope, the calibration‐in‐the‐large and AUC using a prevalence of any PCa of 50% and a standard deviation of the linear predictor of 1.6. With a sample size of 300 men, we are powered to find a width of the 95% CI of 0.49 for the calibration slope, 0.56 for the calibration‐in‐the‐large and 0.09 for the AUC.

## 3 RESULTS

### 3.1 Patient characteristics

A total of 473 patients underwent mpMRI during the inclusion period. Seventy‐seven patients with a prior history of PCa and 72 patients who did not undergo prostate biopsy after mpMRI were excluded, as were 19 patients where mpMRI was not performed prior to biopsy, and two patients with prostatic abscess as the reason for referral. Three hundred and three patients met inclusion criteria; 79% of patients (239/303) were biopsy naïve; 21% (64/303) had undergone previous negative TRUS‐guided systematic biopsies, 16 of these had a subsequent finding of csPCa. PCa was detected in 55% (167/303) of patients, and csPCa in 41% (125/303) of patients; 14% (42/303) had indolent PCa. The median age of included men was 67 (interquartile range [IQR] 62–70) years and the median PSA 8.5 (IQR 6.1–13.9) ng/ml. Median PSA density was 0.17 (IQR 0.11–0.33 ng/ml^2^). DRE was missing in 13.5% (41/303) and suspicious in 36% (94/262). Further clinical data, mpMRI findings and biopsy results in accordance with START criteria are summarized in Tables 1 and 2.^25^

**TABLE 1:** Clinical parameters, mpMRI and biopsy results according to START criteria

	Overall	csPCa	any PCa	no PCa
Men included in analysis, *n* (% of all men)	303 (100)	125 (41)	167 (55)	136 (45)
Median age, year (IQR)	67 (62–70)	68 (64–71)	68 (63–71)	66 (61–69)
Median prebiopsy PSA level, ng/ml (IQR)	8.50 (6.1–13.9)	10.7 (7–28.6)	9.6 (6.7–17.2)	7.5 (5.7–10.9)
Median PSA density (IQR)	0.17 (0.11–0.33)	0.32 (0.19–0.59)	0.24 (0.15–0.49)	0.13 (0.09–0.2)
Median prostate volume, ml (IQR)	46 (33–66)	37 (28—48)	38 (30–51)	59 (42–83)
Suspicious DRE findings (≥T2), *n* (%)	94 (36)	67 (72)	76 (81)	18 (19)
Missing DRE findings, *n* (%)	41 (14)	15 (37)	19 (46)	22 (54)
Previous negative biopsy, *n* (%)	64 (21)	16 (25)	25 (39)	39 (61)
Biopsy naive, *n* (%)	239 (71)	109 (46)	142 (59)	97 (41)
PI‐RADS 1–2 (%)	107 (35)	9 (8)	21 (20)	86 (80)
PI‐RADS 3 (%)	39 (13)	8 (21)	15 (38)	24 (62)
PI‐RADS 4 (%)	54 (18)	28 (52)	40 (74)	14 (26)
PI‐RADS 5 (%)	103 (34)	80 (78)	91 (88)	12 (12)

Abbreviations: csPCa, clinically significant prostate cancer; DRE, digital rectal examination; IQR, interquartile range; mpMRI, multiparametric magnetic resonance imaging; PI‐RADS, Prostate Imaging Reporting and Data System; PSA, prostate‐specific antigen; START, Standards of Reporting for MRI‐targeted Biopsy Studies.^25^

**TABLE 2:** Supplementary mpMRI and biopsy data according to START criteria

Patients biopsied with systematic biopsies, *n* (%)	264 (87)
Patients biopsied with MRGB, *n* (%)	35 (12)
Patients biopsied with transperineal cognitive targeted biopsies, *n* (%)	4 (1)
Median days from mpMRI to biopsy (IQR)	7 (4–10)
Men with PI‐RADS ≥ 3 lesions on mpMRI, *n* (%)	197 (65)
Patients with one PI‐RADS ≥ 3 lesion	142
Patients with >1 PI‐RADS ≥ 3 lesions	55
Number of lesions PI‐RADS ≥ 3	265
Overall PI‐RADS score 1 and 2, *n* (% of all mpMRI)	107 (35)
Number of PI‐RADS score 3 lesions, (% of PI‐RADS lesions ≥ 3)	68 (26)
Number of PI‐RADS score 4 lesions, *n* (% of PI‐RADS lesions ≥ 3)	75 (29)
Number of PI‐RADS score 5 lesions, *n* (% of PI‐RADS lesions ≥ 3)	122 (45)
Biopsies per patient, median (IQR)	14 (9–14)
Systematic TRUS biopsies per patient, median (IQR)	14 (12–15)
Systematic TRUS biopsies per patient with PI‐RADS score 1 and 2, median (IQR)	14 (12–15)
Systematic TRUS biopsies per patient with PI‐RADS ≥ 3, mean (IQR)	14 (12–15)
MR‐guided biopsies per patient, median (IQR)	2 (2–3)
Non‐significant prostate cancers in systematic biopsies, *n* (% of all non‐significant prostate cancers)	35 (84)
Non‐significant prostate cancers in MRGB, *n* (% of all non‐significant prostate cancers)	7 (16)
csPCa in systematic biopsies, *n* (% of all significant prostate cancers)	109 (87)
csPCa in MRGB, *n* (% of all significant prostate cancers)	12 (10)
csPCa in transperineal targeted biopsies, *n* (% of all significant prostate cancers)	4 (3)
Mean number of cores taken for one diagnosis of csPCa from systematic biopsies (*n*)	32
Mean number of cores taken for one diagnosis of csPCa from MRGB biopsies (*n*)	8

Abbreviations: csPCA, clinically significant prostate cancer, IQR, interquartile range; mpMRI, multiparametric magnetic resonance imaging; MRGB, magnetic resonance‐guided biopsy; PCa, prostate cancer; PI‐RADS, Prostate Imaging Reporting and Data System; START, Standards of Reporting for MRI‐targeted Biopsy Studies^25^; TRUS, transrectal ultrasound‐guided.

### 3.2 ERSPC RC 3/4

**FIGURE 1:**
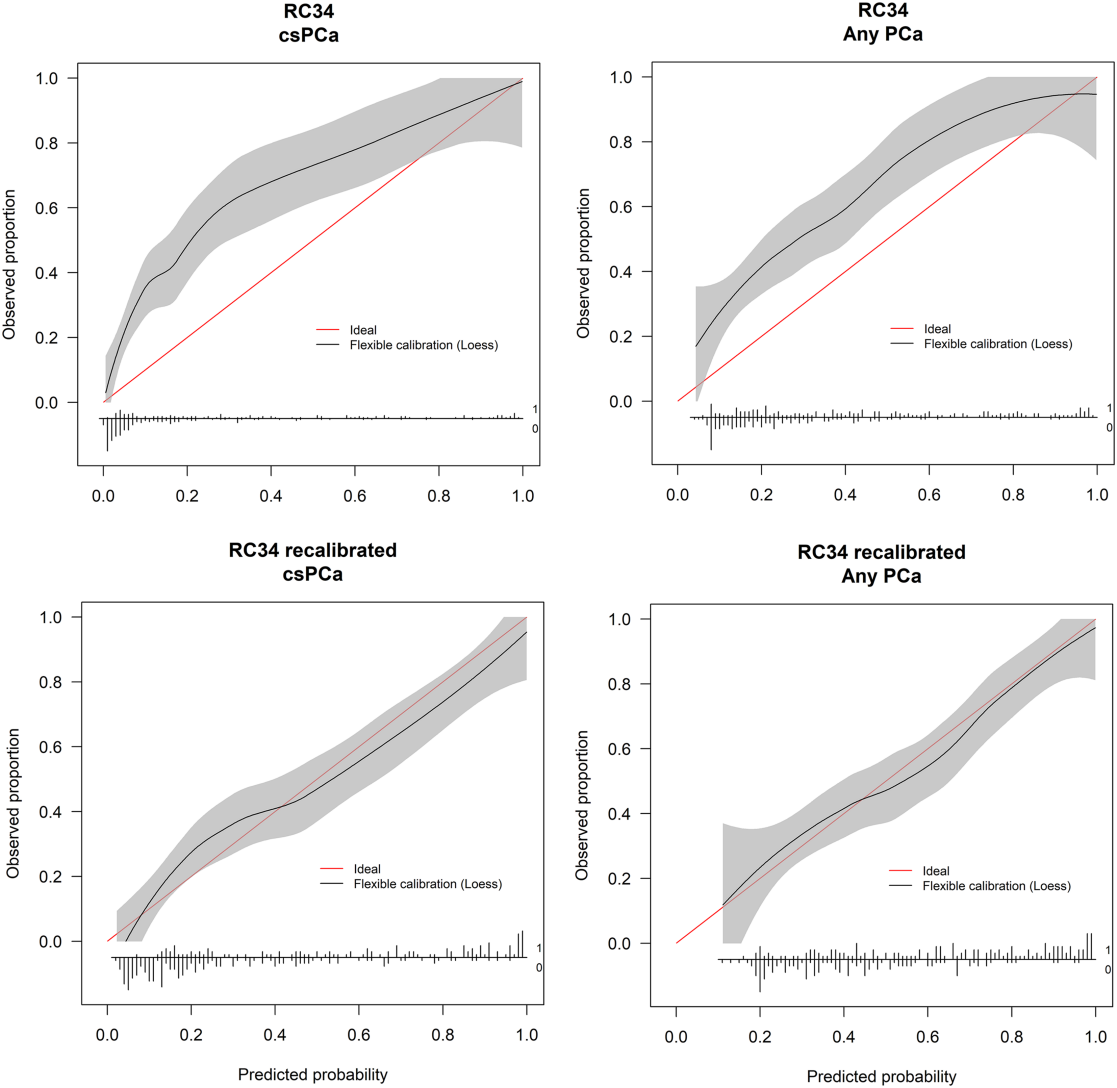
Top: Calibration curve of uncalibrated European Randomized Study of Screening for Prostate Cancer (ERSPC) risk calculators (RC) 3/4 demonstrating the agreement between observed and predicted probabilities for clinically significant prostate cancer (csPCa) (left) and any prostate cancer (PCa) (right) on biopsy. Bottom: Calibration curves after recalibration of ERSPC RC 3/4, demonstrating the agreement between observed and predicted probabilities for csPCa (left) and any PCa (right). The ideal plot shown with a red line. The solid black line shows the relationship between observed and predicted probabilities. Number of patients with and without the condition represented as spikes on the horizontal *x*‐axis

The calibration curve for the un‐calibrated ERSPC RCs 3/4 had an intercept in the large for the detection of any PCa of 1.02 (95% CI 0.75–1.28) and a calibration curve slope of 0.86 (95% CI 0.63–1.12). For csPCa, the intercept in the large was 1.44 (95% CI 1.14–1.73) and the calibration curve slope 0.81 (95% CI 0.63–1.02). Calibration curves before and after recalibration shown in Figure 1.

The AUC for the ERSPC RCs 3/4 was 0.82 (95% CI 0.77–0.87) for the discrimination of csPCa and 0.77 (95% CI 0.72–0.82) for any PCa.

DCA for the detection of csPCa showed a net benefit for the recalibrated ERSPC RCs 3/4 for all threshold probabilities >2% compared with the ‘biopsy all’ approach. For the detection of any PCa, net benefit compared with the ‘biopsy all’ approach was found for threshold probabilities >24% (Figure 3).

### 3.3 MRI‐ERSPC‐RC

**FIGURE 2:**
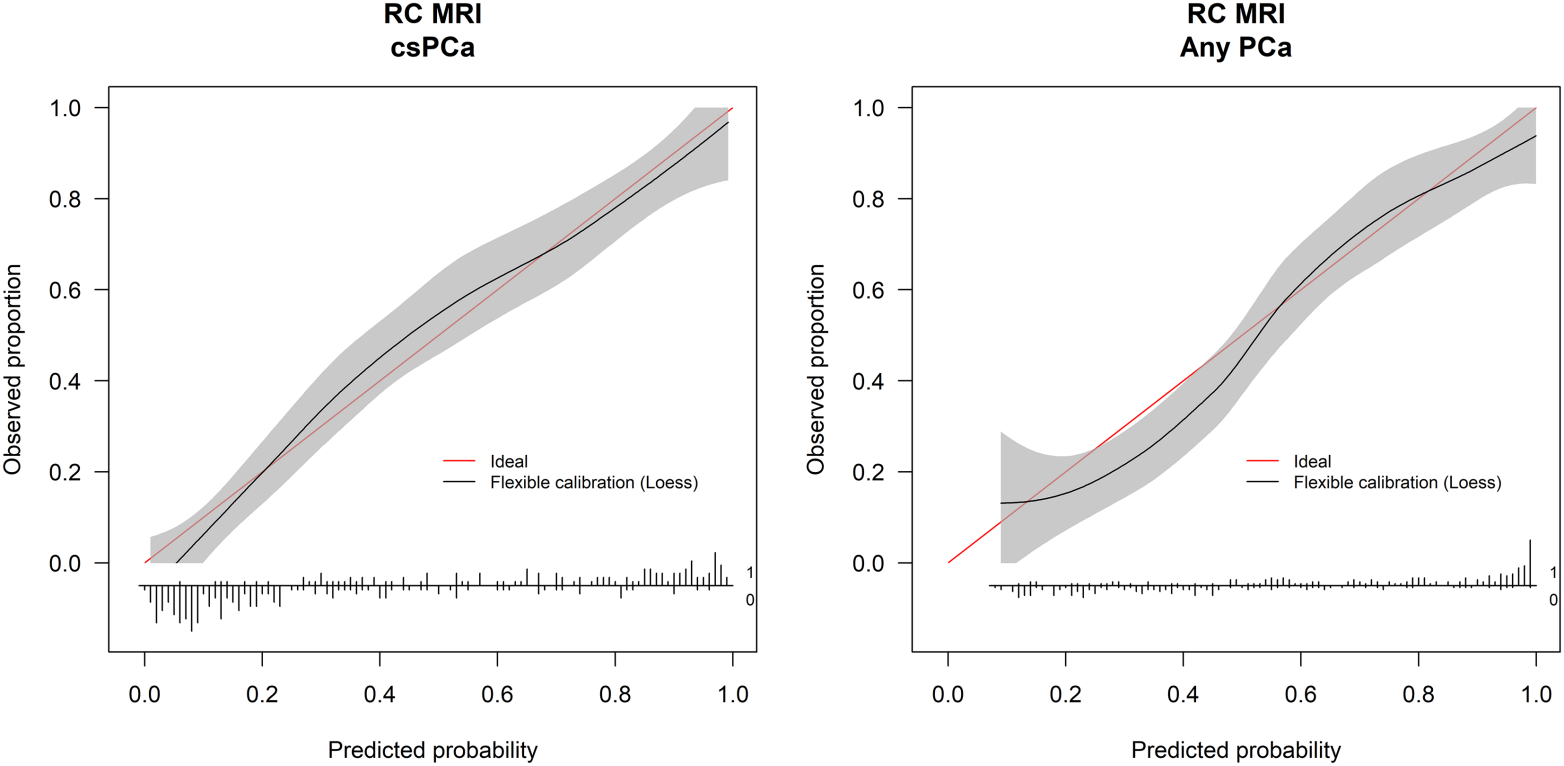
Calibration curves of uncalibrated magnetic resonance imaging European Randomized Study of Screening for Prostate Cancer risk calculators (MRI‐ERSPC‐RCs) demonstrating the agreement between observed and predicted probabilities for clinically significant prostate cancer (csPCa) (left) and any prostate cancer (PCa) (right) on biopsy (no recalibration required) The ideal plot shown with a red line. The solid black line shows the relationship between observed and predicted probabilities. Number of patients with and without the condition represented as spikes on the horizontal *x*‐axis

The calibration curve for the uncalibrated MRI‐ERSPC‐RC for the detection of any PCa had an intercept‐in‐the‐large of −0.26 (95% CI −0.55 to 0.03) and a calibration curve slope of 0.89 (95% CI 0.69–1.12). For the detection of csPCa, calibration of the uncalibrated MRI‐ERSPC‐RC was near perfect, and no recalibration was required. The intercept in the‐large was −0.09 (95% CI −0.40 to 0.21) and the calibration curve slope 1.07 (95% CI 0.85–1.32). Calibration curves were shown in Figure 2.

The AUC for the MRI‐ERSPC‐RC was 0.89 (95% CI 0.85–0.93) for the discrimination of csPCa and 0.85 (95% CI 0.81–0.90) for the discrimination of any PCa.

**FIGURE 3:**
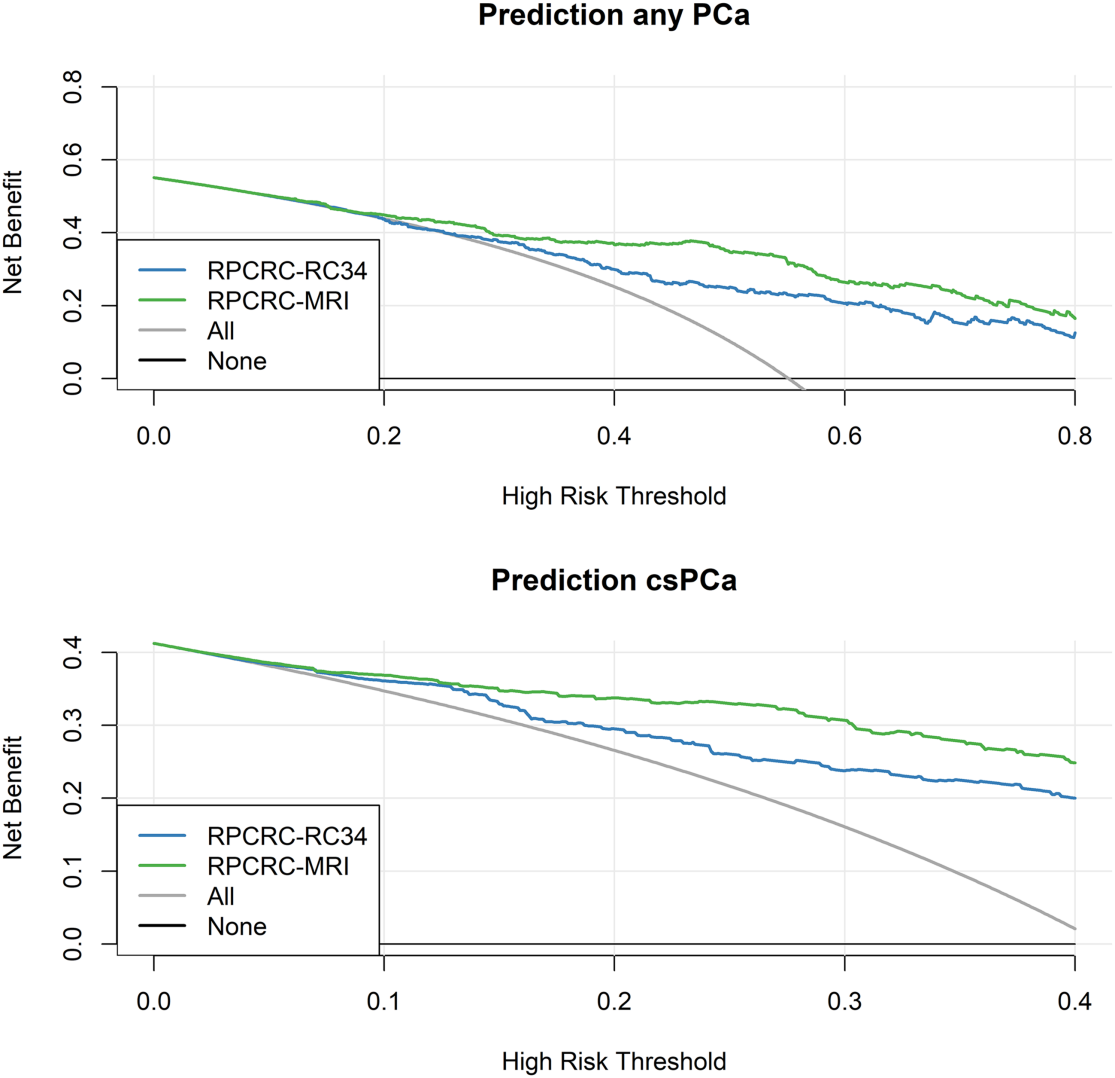
Decision curve analyses demonstrating the net benefit of the recalibrated European Randomized Study of Screening for Prostate Cancer risk calculator (ERSPC‐RC) 3/4 (blue line) and magnetic resonance imaging (MRI)‐ERSPC (green line) risk calculators for the prediction of finding any prostate cancer (PCa) (top) and clinically significant prostate cancer (csPCa) (bottom) on biopsy. Net benefit of ‘biopsy all’ approach (grey line) and ‘biopsy none’—approach (horizontal black line along *x*‐axis)

DCA for the detection of csPCa showed a net benefit for the ERSPC‐MRI‐RC for threshold probabilities > 1% compared with the ‘biopsy all’ approach, as well as compared with the ERSPC RC 3/4. Net benefit was also shown for the MRI‐ERSPC‐RC for the detection of any PCa for threshold probabilities > 8% compared with the ‘biopsy all’ approach, as well as compared with the ERSPC RCs 3/4 (Figure 3).

### 3.4 Biopsies and mpMRI saved versus PCas missed

Table 3 shows the numbers and percentages of mpMRI potentially saved versus csPCa and any PCas detected, had the recalibrated ERSPC RCs 3/4 been applied, at different thresholds to biopsy and standardized to 1000 patients. With a risk threshold of ≥4% of csPCa or 20% of any PCa, 4.6% (14/303) of mpMRI examinations could have been avoided. This would have resulted in 1.8% of PCa cases being missed, (3/167) none of which would have been csPCa. Also shown in Table 3 are the numbers and percentages of biopsy procedures potentially saved after mpMRI versus csPCa and any PCa missed, had the MRI‐ERSPC‐RC been used, at different thresholds to biopsy and standardized to 1000 patients. Using a risk threshold probability of ≥4% of csPCa or 20% of any PCa would have avoided 12.5% (38/303) of biopsies in our cohort, missing 3% (5/167) of cases of any PCa, without any csPCa being missed.

**TABLE 3:** mpMRI and biopsies saved versus prostate cancers detected using different risk thresholds csPCa (GS ≥ 3 + 4) with ERSPC RC 3/4 and MRI‐ERSPC‐RCs in a standardized 1000 men

Risk Threshold	mpMRI performed	mpMRI saved	csPCas detected	Any PCas detected
	*n*	*n* (% of all)	*n* (% of all csPCas)	*n* (% of all PCas)
0% (mpMRI for all patients)	1000	0	410 (100%)	551 (100%)
4% csPCa or 20% any PCa	954	46 (4.6%)	410 (100%)	541 (98.2%)
10% csPCa or 20% any PCa	845	155 (15.5%)	407 (99.2%)	514 (93.4%)
15% csPCa or 20% any PCa	729	271 (27.1%)	387 (94.4%)	475 (86.2%)
20% csPCa or 20% any PCa	617	383 (38.3%)	358 (87.2%)	429 (77.8%)

Risk Threshold	Men biopsied	Biopsies saved	csPCas detected	Any PCas detected
	*n*	*n* (% of all)	*n* (% of all csPCas)	*n* (% of all PCas)
0% (biopsy all patients)	1000	0	410 (100%)	551 (100%)
4% csPCa or 20% any PCa	875	125 (12.5%)	410 (100%)	534 (97%)
10% csPCa or 20% any PCa	766	234 (23.4%)	407 (99.2%)	518 (94%)
15% csPCa or 20% any Pca	690	310 (31%)	397 (96.8%)	495 (89.8%)
20% csPCa or 20% any Pca	614	386 (38.6%)	390 (95.2%)	482 (87.4%)

Abbreviations: csPCa, clinically significant prostate cancer; ERSPC RC, European Randomized Study of Screening for Prostate Cancer risk calculator; mpMRI, multiparametric magnetic resonance imaging; PCa, prostate cancer.

Using the MRI‐ERSPC‐RCs, eight patients with PI‐RADS ≥ 3 findings had a <4% calculated probability of csPCa or 20% of any PCa, none of whom had csPCa on biopsy. Twenty‐two patients with PI‐RADS ≥ 3 had a <10% calculated probability of csPCa or 20% of any PCa; one of these had csPCa on biopsy.

### 3.5 Sensitivity analyses

We performed sensitivity analyses by removing patients who received targeted biopsies by MRGB (*n* = 35) and transperineal cognitive targeted biopsies (*n* = 4); this did not alter the RC's discriminative ability significantly (data not shown).

## 4 DISCUSSION

Risk‐based selection of patients who require prostate mpMRI and biopsy for suspected PCa is already part of urological practice but can be improved considerably by using multivariable RCs. In a recent PSA‐based screening study by Wallström et al., 75% of mpMRI were negative,^26^ as were 35% of mpMRI in the current cohort despite its high cancer prevalence. This emphasizes the need for risk stratification before mpMRI. As all patients in our cohort received both upfront prostate mpMRI and prostate biopsy, a substantial number of patients could be identified in whom a costly and time‐consuming mpMRI could be omitted. If used as an initial assessment tool for identifying patients who warrant further investigation with mpMRI, the recalibrated ERSPC‐RCs 3/4 at a ≥10% of csPCa or 20% probability of any PCa threshold to biopsy would save 15.5% (47/303) of all mpMRI investigations in our cohort, at the cost of missing only 0.8% (1/125) of all csPCa, equivalent to csPCa being missed in 2% (1/47) of omitted mpMRI scans.

For patients who undergo mpMRI, using the MRI‐ERSPC‐RCs with a ≥10% risk of csPCa or 20% risk of any PCa threshold to biopsy would have reduced the number of biopsies by 23.4% (71/303). This approach would detect 99.2% (124/125) of all csPCa and 94% (160/167) of all PCa cases. Despite the high cancer prevalence in our cohort, this is comparable to the findings of previous studies.^6^, ^14^, ^27^, ^28^

Choosing an optimal probability threshold for when to recommend mpMRI or prostate biopsy is difficult,^23^ as this will differ on a case by case basis and depend on patient comorbidities, age, life‐expectancy, as well as urologist and patient preferences.^8^ For this reason, we consider a range of threshold probabilities, to cover most clinical scenarios where mpMRI or biopsy could be warranted. Based on the current data however, it would not seem justified to recommend mpMRI or biopsy below a 4% risk of csPCa or 20% risk of any PCa as calculated by the ERSPC 3/4 RCs or the MRI‐ERSPC‐RCs. Below this threshold, the patient would not be at risk of harbouring csPCa, and performing mpMRI or biopsy would only add cost, risk of complications and the risk of uncovering an indolent PCa.

The calibration curves for the uncalibrated ERSPC RCs 3/4 show excellent performance but also highlight the need for adjustment for differences in prevalence when applying RCs to new populations. The development and validation study populations differ in their geographic origins and in their composition. Where the ERSPC RCs 3/4 were developed based on the data of a screening population (3624 men in the first screening round for RC3, and 2896 men in the second screening round for RC4 of the ERSPC in Rotterdam),^5^ the current data are from a clinical referral cohort at a Norwegian university hospital. The ERSPC RCs 3/4 were developed in a cohort of men with PSA 0.4–50 ng/ml, prostate volume between 10 and 110 ml and age between 50 and 75,^27^ although in our study, no exclusions were made based on age, PSA or prostate volume. Men in the ERSPC RC3 and our cohort had comparable median age and proportion of suspicious DREs, although prebiopsy PSA levels were higher for the current cohort (65.8 years, 35.4% and 4.3 ng/ml vs. 67 years, 36% and 8.5 ng/ml). Finally, the original study by Roobol et al. used a lateralized sextant biopsy scheme, although in our study, an extended core biopsy pattern was applied, with a median of 14 (IQR 12–15) biopsies per patient. These differences could contribute to the systematic underestimation of probabilities of both any PCa and csPCa of the uncalibrated ERSPC RC 3/4, adjusted for by recalibration.

The original MRI‐ERSPC‐RCs was nearly perfectly calibrated to our cohort for csPCa and showed very good calibration for any PCa. The MRI‐ERSPC‐RCs were developed from clinical data from 961 men with a clinical suspicion of PCa. Patients received mpMRI and subsequent TRUS‐guided systematic biopsies and/or targeted biopsies (MRGB, MRI‐TRUS fusion and cognitive fusion) in case of positive mpMRI (PI‐RADS > 2 using PI‐RADS v1).^14^ The development cohort and our validation cohort had very similar age, PSA, prostate volume, suspicious DRE and PSA density characteristics (development cohort median 66.0 years, 8.7 ng/ml, 49.7 ml, 39%, 0.17 ng/ml^2^ vs. the current study 67 years, 8.5 ng/ml, 46 ml, 36%, 0.17 ng/ml^2^). Cohorts differed in their rates of positive mpMRI, 82% having PI‐RADS ≥ 3 in the development cohort versus 65% (197/303) in our validation cohort, and in that, 83% received MRI‐targeted biopsies in the development cohort while 87% were biopsied with TRUS‐guided systematic biopsies only in our cohort. In a recent Cochrane review, targeted biopsies (MRGB or MR/US fusion) had higher sensitivity (0.80, 95% CI 0.69–0.87) for detecting ISUP GG ≥ 2 PCa than systematic biopsies (0.63, 95% CI 0.19–0.93), using template‐guided biopsies as the reference standard.^29^ Given the similarities between cohorts, but different biopsy techniques, the perfect agreement with the uncalibrated MRI‐ERSPC‐RCs was surprising. This could in part be explained by the fact that treating urologists were not blinded to the findings of mpMRI and therefore had the option of performing cognitively focused biopsies in addition to systematic biopsies. There was however no difference in the number of TRUS‐guided biopsies obtained in patients with positive mpMRI (PI‐RADS ≥ 3) (median = 14, IQR 12–15) compared with those with negative mpMRI (PI‐RADS ≤ 2). Another explanation could be a higher PCa prevalence in our cohort than in the development cohort. Alberts et al.^14^ found 51% to have any PCa and 36% csPCa. In our cohort, the observed prevalence of PCa was 55% (167/303) and of csPCa 41% (125/303) but could nonetheless have been even higher, as a strict reference test such as template mapping biopsies was not performed in this validation study.

In the development study of the original ERSPC RCs 3/4,^27^ predictive accuracy was reported with an AUC of 0.79 (0.71–0.87) for the discrimination of csPCa and 0.70 (0.63–0.76) for any PCa, which is similar to the findings of this, as well as previous, validation studies.^6^, ^8^, ^28^ The MRI‐ERSPC‐RC3 was originally described with an AUC for csPCa of 0.84 (95% CI 0.81–0.88) for biopsy‐naïve men and the MRI‐ERSPC‐RC4 for previously biopsied men with an AUC of 0.85 (95% CI 0.81–0.89), which is lower than we found in this external validation, although with overlapping CIs.

A net benefit was found for the use of the recalibrated MRI‐ERSPC‐RCs for the detection of csPCa for risk thresholds as low as >1%, which was lower than that of the development study of Alberts et al.,^14^ where a net benefit was only seen above the ≥5% risk threshold. The net benefit of using both the ERSPC and ERSPC‐MRI RCs is higher for the prediction of csPCa than of any PCa. This reflects clinical practice in the current era of active surveillance, where the decision to biopsy should be driven by the likelihood of finding csPCa, rather than an indolent cancer.

A small number of patients had positive mpMRI (PI‐RADS ≥ 3) and low probabilities of csPCa and PCa using both the uncalibrated and the recalibrated MRI‐ERSPC‐RCs. Targeted biopsies are currently recommended in patients with positive mpMRI (PI‐RADS ≥ 3).^13^ In our data, csPCa was found in 21% of patients with PI‐RADS 3 on mpMRI, which could reflect the high cancer prevalence in the study population. Nonetheless, as our findings and the previously reported low specificity of mpMRI for detection of csPCa of 0.37 suggest,^29^ many patients with PI‐RADS ≥ 3 on mpMRI do not have csPCa and multivariable risk‐based selection of patients for biopsy even in these patients seems warranted. Without targeted biopsies, this delineation was not possible in our cohort, but future works may better identify patient subgroups with PI‐RADS ≥ 3 where biopsy may be deferred.

The current work represents a real‐world recalibration and validation of the ERSPC RC 3/4 and MRI‐ERSPC‐RCs to a Norwegian university hospital cohort. Patients were not excluded based on age, PSA or prostate volume, and patients with both systematic and targeted biopsy methods were included without this altering the discriminative abilities of the RCs in question. Other strengths of our study are that only the PI‐RADS v2.0 reporting scheme was used, which may perform slightly better than the previous PI‐RADS v1.0 version.^30^

Our study has some limitations. Most patients were biopsied with systematic biopsies, rather than targeted biopsies, limiting the utility of the MRI‐ERSPC‐RCs in positive mpMRIs. This however reflects clinical practice at our institution during the inclusion period. Seventy‐two patients were excluded from analysis as no biopsy was taken after mpMRI. It could not be determined with certainty if these patients harboured PCa at the time of assessment, nor how it would have affected results had biopsy been performed. This is a limitation of this as well as of other similar works.^6^, ^14^ Cancer prevalence was high in our cohort compared to in other reports.^6^, ^14^ Prevalence may have been falsely elevated by some selection bias unknown to the authors or may reflect the underlying cancer prevalence in the study cohort. If the cancer prevalence in the current calibration cohort is higher than in the population where the RCs will be used, the recalibrated RCs will overestimate cancer risk, leading to fewer mpMRIs and biopsies saved, but not to more cases of PCa or csPCa being missed. The clinical benefit of omitting mpMRI or biopsy in men with a low calculated risk of PCa by RCs has to our knowledge not been defined. Subgroup analyses on biopsy‐naïve and previously biopsied patients could not be performed due to a low number of patients with prior negative biopsies. Lastly, our study was retrospective, and from a single centre, which may put generalizability to other Scandinavian centres into question, however, this study population likely mirrors the general Scandinavian population better than the development European cohorts.

Despite these limitations, the recalibrated ERSPC RC 3/4 and MRI‐ERSPC‐RCs provide a well‐founded basis to omit both mpMRI and prostate biopsy in patients with low calculated risk of PCa at little cost in a contemporary Norwegian setting.

## 5 CONCLUSIONS

Use of the herein recalibrated and validated ERSPC RC 3/4 and MRI‐ERSPC‐RCs can result in considerable reduction both of upfront mpMRI examinations and of unnecessary systematic biopsies. The use of pre‐biopsy RCs is recommended in current international clinical practice guidelines, and the current findings facilitate their implementation in Scandinavian countries.

## FUNDING INFORMATION

Funding from the Research Council of Norway was received, Grant no. 295013, funding for research position for author P.D., Department of Clinical and Molecular Medicine, The Norwegian University of Science and Technology NTNU, NO‐7491 Trondheim, Norway.

## CONFLICT OF INTEREST

ME received grant from The liaison Committee between the Central Norway Regional Health Authority and the Norwegian University of Science and Technology (Grant Number 90265300); other authors declared none.

## AUTHOR CONTRIBUTIONS

*Study concept and design*: Monique J. Roobol, Helena Bertilsson, Tone Frost Bathen, Mattijs Elschot, Petter Davik and Sebastiaan Remmers. *Data acquisition*: Petter Davik. *Statistical analysis*: Sebastiaan Remmers and Petter Davik. *Drafting of the manuscript*: Petter Davik and Sebastiaan Remmers. *Critical revision of manuscript*: Helena Bertilsson, Tone Frost Bathen, Mattijs Elschot, Monique J. Roobol and Sebastiaan Remmers.
